# Comparison of the Effects of Two Auditory Methods by Mother and Fetus on the Results of Non-Stress Test (Baseline Fetal Heart Rate and Number of Accelerations) in Pregnant Women: A Randomized Controlled Trial 

**Published:** 2016-03

**Authors:** Roghaie Khoshkholgh, Tahereh Keshavarz, Zeinab Moshfeghy, Marzieh Akbarzadeh, Nasrin Asadi, Najaf Zare

**Affiliations:** 1 Department of Midwifery, Islamic Azad University, Firuzabad Branch, Firuzabad, Iran; 2 Department of Midwifery, Community Based Psychiatric Care Research Center, School of Nursing and Midwifery, Shiraz University of Medical Sciences, Shiraz, Iran; 3 Maternal–Fetal Medicine Research Center, Department of Midwifery, School of Nursing and Midwifery, Shiraz University of Medical Sciences, Shiraz, Iran; 4 Department of Obstetrics and Gynecology, School of Medicine, Shiraz University of Medical Sciences, Shiraz, Iran; 5 Department of Biostatistics, School of Medicine, Infertility Research Center, Shiraz University of Medical Sciences, Shiraz, Iran

**Keywords:** Auditory, Fetus, Non Stress Test, Mother

## Abstract

**Objective:** To compare the effects of two auditory methods by mother and fetus on the results of NST in 2011-2012.

**Materials and methods:** In this single-blind clinical trial, 213 pregnant women with gestational age of 37-41 weeks who had no pregnancy complications were randomly divided into 3 groups (auditory intervention for mother, auditory intervention for fetus, and control) each containing 71 subjects. In the intervention groups, music was played through the second 10 minutes of NST. The three groups were compared regarding baseline fetal heart rate and number of accelerations in the first and second 10 minutes of NST. The data were analyzed using one-way ANOVA, Kruskal-Wallis, and paired T-test.

**Results:** The results showed no significant difference among the three groups regarding baseline fetal heart rate in the first (p = 0.945) and second (p = 0.763) 10 minutes. However, a significant difference was found among the three groups concerning the number of accelerations in the second 10 minutes. Also, a significant difference was observed in the number of accelerations in the auditory intervention for mother (p = 0.013) and auditory intervention for fetus groups (p < 0.001). The difference between the number of accelerations in the first and second 10 minutes was also statistically significant (p = 0.002).

**Conclusion:** Music intervention was effective in the number of accelerations which is the indicator of fetal health. Yet, further studies are required to be conducted on the issue.

## Introduction

Evaluation of fetal health is one of the main components of prenatal care. In fact, evaluation of fetal health aims at identification of high-risk fetuses for prevention of permanent damage or death, identification of healthy fetuses to avoid unnecessary interventions (1, 2). Thus, achieving a specific program for diagnosis of asphyxia during pregnancy has always been a matter of concern for researchers (3).

A new phenomenon has evolved in modern midwifery medicine which predicts fetal health Besides, tests such as Non-Stress Test (NST), Biophysical Profile (BPP), and Oxytocin Challenge Test (OCT) have been considered as the major components of midwifery care (4-6). The main goal of monitoring is prevention of fetal and infantile complications, particularly before fetal death (5).

Tsubokora et al. (2002) stated that reduction in fetal movements resulted from damage to fetus’ brain or even its death. Nevertheless, this cannot always hold true and the reasons for these changes should be evaluated accurately. Since fetal movements are naturally followed by a considerable increase in fetal heart rate, assessment of fetal heart rate using NST can provide more precise results (6).

NST is in fact a test of fetus status and an indirect evaluation of fetus’ oxygenation status. In contrast to counting the number of fetal movements by the mother, NST is performed by trained personnel using medical equipment. NST is based on increase in heart rate of a fetus without acidosis (hypoxia or neurological weakness) which occurs due to fetal movements. In this condition, blood flow speed is assessed instead of blood volume. Various long-term experiments have shown that blood flow speed is varied in different physiological and pathological conditions (4).Up to now, numerous methods have been proposed for conversion of non-reactive to reactive tests. These methods include prolonging the test period, moving the fetus, feeding the mother with glucose, using halogen for the mother, and acoustic stimulation of mother and fetus before the test (7-9). A few studies conducted on moving the fetus have revealed that it had some effects on fetal movements, but not on other test parameters, such as fetal cardiac response. In addition, utilization of halogen had some impacts on fetal heart rate accelerations, but its disadvantages were not determined. Besides, consumption of glucose, either in form of fruit juice or glucose solution, had no effects on the test results, if not a contraindication (10-13). Nonetheless, most fetal movements were found to respond to acoustic stimulation. It has been suggested that application of external sounds of 100-105 dB on mother’s abdomen for 3 seconds could be repeated for 3 times. Evidence has indicated that mother’s exposure to vibroacoustic stimulation could induce fetal movements (14).

Although no direct neuronal link is there between mother and fetus, mother’s experiences can produce a physiological and neurochemical cascade which stimulates the uterus environment and leads to fetal response directly or indirectly (15).

A large number of researches have disclosed that music intervention in the field of health could decrease worries, pain, and stress (16, 17). For instance, it has been reported that classical music with soft rhythms could reduce heart rhythm and blood pressure, deepen breathing, decrease stress, and reduce the need for medications (18, 19).

In a study in Turkey was revealed a strong negative relationship between mother’s stress and fetal heart rate accelerations. They also reported that listening to music while performing the test increased fetal movements and heart rate accelerations (20).

The findings of the studies showed no evidence of damage to brainstem or auditory nerve. Thus, it seems that the potential clinical effect of this method overshadows its theoretical risk (21-22).

Overall, NST is one of the methods used for evaluation of fetal health through the last weeks of pregnancy. In this method, number of accelerations is indicator of fetal health (1, 2). In addition, acoustic stimulation is a proper method for conversion of non-reactive to reactive tests (23). Besides, music intervention can have potential effects on the mother (24) and fetus that also has the auditory sense can respond to the complex sound of music (14). Therefore, the present study aims to compare the effects of two auditory methods by mother and fetus on NST parameters (baseline fetal heart rate and number of heart rate accelerations) in the pregnant women referring to clinics of the selected hospitals of Shiraz University of Medical Sciences.

## Materials and methods

**Figure1:**
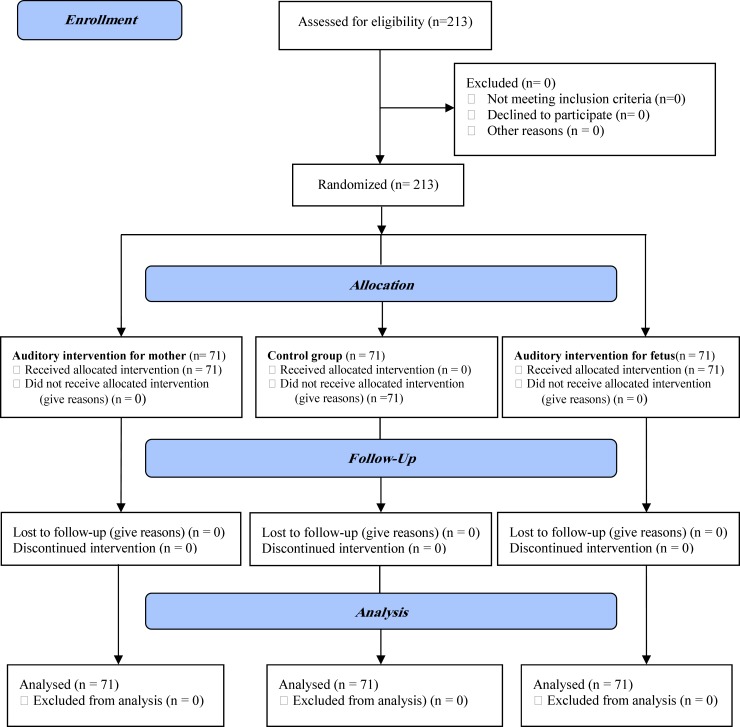
CONSORT Diagram

This was a single-blind randomized controlled trial conducted from May 2011 to March 2012. The study population consisted of 213 pregnant women with between 18 and 35 years old with gestational age of 37-41 weeks who were in prenatal clinic and referred to Shiraz Hafez Hospital, Iran. Based on previous research (20) considering 𝑑 = 1.2, 𝛼 = 0.05, 1 − 𝛽 = 0.90, and the following formula, a 213-subject sample size (71 subjects in each group) was determined for the study (Figure 1).

$n=\frac{2{\left(z_{1-\frac{\alpha }{2}}+z_{1-\beta }\right)}^{2}\sigma^{2}}{d^{2}}$

The inclusion criteria of the study were singleton pregnancy, not suffering from pregnancy complications such as blood pressure disorders, insulin-requiring diabetes mellitus, IUGR fetus, preterm premature rupture of membranes, multiple births, intrauterine fetal death, lethal fetal anomalies, known cardiovascular anomalies, oligohydramnios, and encephalitis, not suffering from hearing loss or hearing impairment, not having chronic diseases, not using analgesics and sedatives, not having severe physical activity 2 hours before beginning of the study, passage of at least 1.5 hours from the last meal, and not smoking or using drugs.

Then provided the study participants with information about the aim and the process of the study and invited them to read and sign the written consent form of the study.

The music intervention was performed for the mother in the first intervention group and for the fetus in the second one, while the third group was considered as the control.

Music intervention for the mother was performed through the second 10 minutes of NST. The utilized music was an instrumental duet by Paganini (violin and guitar) and Chopin (piano) which was entered into a computer system and was played to the mothers using k44 headphones. It should be noted that actions, such as drinking juice and talking with the patient, were not done for reduction of mothers’ stress. Additionally, the test was performed by a single individual in each center. The intervention was conducted at morning and evening shifts so as to decrease the effect of time on the test results.

After determining the location of fetus’ head, music intervention for fetus was performed at 95 dB for 15 seconds in the second 10 minutes of the test. The music was played by a computer system through Adobe Audition software which is used for determining sound intensity and music dynamic peak. The fetus was exposed to music through a headphone with noise cancelling structure which is used to eliminate the surrounding sounds. In order to ensure that the music was played for 15 minutes, the earphone was put on the mother’s abdomen for 20 seconds with the first 5 minutes being soundless. This was done to make sure that the device was on and that the music was played at due time. During these 15 seconds, a piece of music with three piu fortissimos was played for the fetus. In doing so, a section of the music “So said Zoroaster” was recomposed and the intervals among its piu fortissimos were determined. The researchers found no side effects resulting from acoustic stimulation. Although higher sound levels were used in the previous studies, acoustic stimulation levels were modified in the present research (21, 22).

After acoustic stimulation, fetal monitoring was done and fetal electrocardiogram was recorded by Analogic Lite-TM device. Besides, NST strips were divided into two 10-minute parts using Photoshop software. The provided reports were interpreted by a perinatologist who was unaware of the intervention process regarding the number of accelerations and baseline fetal heart rate.

In case of non-reactive rests, the procedure was repeated and if similar results were obtained again, the patient was referred for BPP or Contraction Stress Test (CST) according to the hospital’s protocol.

After all, one-way ANOVA followed by Tukey HSD test and Kruskal-Wallis test were used to compare the study groups’ demographic characteristics. Besides, paired T-test was used to compare the study groups before and after the intervention. All the analyses were performed at 90% Confidence Interval (CI) and α = 0.05.

***Ethical considerations***

This study was approved by the Ethics Committee of Shiraz University of Medical Sciences (with number: 90-5813).The study objectives were explained to the patients before they entered the study, and an informed consent was obtained from all.

## Results

The mean age of the three study groups was between 26.5 and 27 years and the results of one-way ANOVA showed no significant difference among the three groups in this regard (p = 0.675). Also, no significant difference was found among the three groups with respect to Body Mass Index (BMI) (p = 0.452), gestational age (p = 0.224), and gravidity (p = 0.844).

The results of Kruskal-Wallis test revealed no significant difference among the three groups regarding baseline fetal heart rate in the first (p = 0.945) and second (p = 0.763) 10 minutes of the test (Table 1). Also, no significant difference was found among the three groups concerning the mean of accelerations in the first 10 minutes of the test (p = 0.763) (Table 1). Out of the 213 mothers under study, 100, 59, 37, 12, and 5 ones had no, 1, 2, 3, and 4 accelerations, respectively in the first 10 minutes . However, the results showed a significant difference among the study groups regarding fetal heart rate accelerations in the second 10 minutes of the test (p = 0.020). According to the results of Tukey HSD test, the difference between the control and fetus groups, but not between the control and mother groups, was statistically significant (p = 0.034) (Table 1). Out of the 213 mothers under study, 81, 59, 52, 14, 7, and 3 ones had no, 1, 2, 3, 4, and 5 accelerations, respectively in the second 10 minutes.

In the music intervention for mother group, no significant difference was found between baseline fetal heart rates in the first and second 10 minutes of the test. However, a significant difference was observed in the number of accelerations (p = 0.013) (Table 2).

**Table 1:** Results of fetal Baseline heart rate and acceleration of the three groups in the first 10 minutes of non-stress testing in both interventions and control groups (n = 71)

**Variable**	**Group**	**mean ± SD**	**p**
Baseline of fetal heart rate in first 10 minutes	Mother	138.1 ± 9.2	0.945٠
	Fetus	138 ± 8.3	
	Control	138.6 ± 9.5	
Baseline of fetal heart rate in second 10 minutes	Mother	138 ± 9.1	0.763
	Fetus	138.4 ± 9	
	Control	138.7 ± 8.9	
Acceleration in first 10 minutes	Mother	0.8 ± 0.9	0.812
	Fetus	0.9 ± 1	
	Control	1 ± 1.2	
Acceleration in second 10 minutes	Mother	1.1 ± 1.2	0.020
	Fetus	1.4 ± 2.2	

**Table 2:** Comparing the results of non-stress tests in the first and second 10 minutes of the three groups

			**Mother intervention**	**Fetus intervention**	**Control**
Baseline of fetal heart rate	First 10 minutes	mean ± SD	138 ± 8.3	138.1 ± 9.2	138.6 ± 9.5
	Second 10 minutes	mean ± SD	138.4 ± 9	138 ± 9.1	137.7 ± 8.9
	p		≥ 0.05	≥ 0.05	≥ 0.05
Acceleration	First 10 minutes	mean ± SD	0.9 ± 1	0.8 ± 0.9	1 ± 1.2
	Second 10 minutes	mean ± SD	1.4 ± 1.2	1.1 ± 1.2	0.9 ± 1.1
	p		0.013	0.001	≥ 0.05

In the music intervention for fetus group also, no significant difference was observed between baseline fetal heart rates in the first and second 10 minutes of the test. However, a significant difference was observed in the number of accelerations (p < 0.001) (Table 2). The difference between the number of accelerations in the first and second 10 minutes was also statistically significant (p = 0.002) (Table 3).

**Table 3:** Compare the difference in the accelerations and beat to beat variability in both interventions and control groups

	**Acceleration difference ** **(mean ± SD)**	**p**
Control	0.03 ± 0.92	0.002
Mother	0.28 ± 0.93	
Fetus	0.53 ± 1.01	

## Discussion

The findings of the present study revealed no significant difference among the three groups regarding baseline fetal heart rate in the first and second 10 minutes of the test. In contrast, a study in Turkey to assess the effect of mother’s worries and music on fetal movements and heart rate pattern and reported that executing auditory intervention for mothers increased baseline fetal heart rate (25). In that study, 201 pregnant women with gestational age of 36-41 weeks who had no chronic pregnancy complications were randomly divided into an intervention (n = 96) and a control (n = 105) group. Besides, the intervention was conducted using classical instrumental music (Haydn, Mozart, and Beethoven), Turkish local music, and Turkish folk music. It should be noted that the mothers could give their opinions about the pieces of music and control the volume. According to the results, the mean gestational age was 38.1-38.5 weeks in both groups and the two groups were similar with respect to demographic characteristics, which is in line with the findings of the current study. Moreover, the scores of NST were similar before the test, but the control group participants showed significantly higher scores compared to the intervention group after the test. Thus, it was concluded that the process of NST was stressful for mothers and music intervention increased baseline fetal heart rate and number of accelerations in the intervention group compared to the control group (p < 0.001). In the present study, on the other hand, a piece of international classical music was used with which the study participants were not familiar. Furthermore, Kafali et al. executed the intervention all through the test, while we started the intervention from the second 10 minutes so that in addition to comparing each group before and after the intervention, we could compare the two groups at intervention and non-intervention time points and had basic information about fetal heart patterns within the first 10 minutes of the test.

In a study carried out a research in the U.S. to evaluate fetal response to increase in mother’s relaxation during pregnancy. That study was conducted on the mothers with above 32 weeks of gestation who had no pregnancy complications and relaxation was induced through mental imagery and music. The study results showed that music intervention decreased baseline fetal heart rate (15). In the present study, however, paying classical music to mothers had no impacts on baseline fetal heart rate.

The findings of the current study revealed a significant difference between the control and the fetus group regarding the number of accelerations in the second 10 minutes of the test (p = 0.034). Nonetheless, no significant difference was found between the mother and fetus groups as well as between the mother and control groups in this respect. These findings were on the contrary to those obtained by Kafali (25) who reported that playing music to mothers affected both baseline fetal heart rate and accelerations.

In a study conducted a study entitled “fetal response to music and sound” to assess the auditory effects of music and sound on fetal behaviors. In that study, 20 pregnant women at 37-40 weeks of gestation who had no pregnancy complications were randomly divided into two groups. The 10 participants in the intervention group were exposed to music and sound through single earphones, while the rest of the participants were considered as the control group. The intervention group participants were exposed to a piece of Spanish music played with guitar for 15 seconds. The music was played at 80 dB and the maximum sound pressure was considered at 94-105 dB. Using ultrasound, the two groups were compared regarding fetal heart pattern before and after the intervention. The results showed no significant difference between the two groups concerning fetal heart pattern before the intervention. After the intervention, however, the two groups were significantly different with respect to the number of accelerations (26). These results were in agreement with those of the current study. Yet, in the present study, each group consisted of 71 participants and the music intervention was performed using a recomposed piece of music at 95dB which had 3 piu fortissimos. This lower acoustic power was selected to keep the fetus safe from any probable risks.

In a study assessed the effect of vibroacoustic stimulation on fetus in 486 mothers above 32 weeks of gestation with spontaneous labor. The results of that study were consistent with those of the current one in the increase in the number of accelerations in response to acoustic stimulation of the fetus. Nonetheless, the present study was performed on the pregnant women with 37-41 weeks of gestation when the fetus is capable of responding to music which is a complex sound. Besides, since the study aimed at comparison of maternal-fetal responses to music, it involved the mothers who had no labor contractions or pains. In this way, the fetus group was under the same condition as the intervention for mothers group where it was attempted to keep the mother calm through playing music (27).

The findings of the current study revealed no significant difference between the first and second 10 minutes of the test regarding baseline fetal heart rate, but the difference in the number of accelerations was statistically significant (p = 0.013). This was relatively consistent with the results obtained by Kafali et al. (2010). That study compared the intervention group to the controls, while the present one compared each study group before and after the intervention. In contrast to Kafali’s study, the music intervention for mother had no effects on baseline fetal heart rate in our study.

Fetal heart acceleration is defined as a detectable abrupt increase in baseline fetal heart rate which is in almost all cases accompanied by fetal movements. These accelerations are quite reassuring and confirm that the fetus is not acidemic at that moment (1). Evidence has shown that music has a critical effect on the autonomic nervous system and leads to suppression of sympathetic and parasympathetic activities, eventually lowering heart rate, increasing cardiac output, activating peripheral vasodilators, and lowering blood pressure. It also affects the respiratory system and deepens breathing which result in better oxygenation and facilitate relaxation (20, 28). Through appropriate oxygenation, fetus is able to move its body. On the other hand, relaxing music can suppress severe sympathetic effects, such as worry, tachycardia, and tachypnea. Of course, these effects are induced by hormones and neurotransmitters such as acetylcholine, endogenous opioids such as endorphin and enkephalin, and mono amines such as serotonin which play key roles in feeling of happiness, pain, calmness, and well-being. Music also covers the stressful sounds of the environment and distracts the mind from pain and stress (20). Therefore, increase in the number of accelerations might have resulted from mother’s relaxation and lower stress and increase in fetal movements indicated by increase in baseline fetal heart rate in NST. On the other hand, lack of change in fetal heart rate could be due to the reduction in mother’s sympathetic system activity and hear rate. Previous researches have also demonstrated that music therapy and relaxation decreased mother’s sympathetic system activity and improved her cardiovascular function (29, 30).

Considering improvement in fetus’ cardiac status, it should be mentioned that fetus’ status is to a great extent dependent on mother’s status (31). Ying et al. evaluated the impact of music therapy on reduction of anxiety in 120 pregnant women. These women listened to their favorite music for 30 minutes through three days. The study results revealed that music led to reduction of women’s anxiety, improvement of maternal physiological response, and regulation of fetal heart rate (32).

One of the limitations of the present study was lack of space in the study centers, so that the researchers had to use the NST room. Of course, special headphones which could decrease the surrounding sounds were utilized to eliminate the effect of this factor. Moreover, the manipulations of the individuals responsible for performance of NST could affect the fetus’ consciousness, which was controlled by performing the test by a single individual (the researcher). It should also be noted that the mothers who were unwilling to cooperate were excluded from the study.

## Conclusion

The results of this study showed a significant difference among the three groups regarding the number of fetal heart accelerations. Also, a significant difference was observed in the number of accelerations in the intervention for mother group before and after the intervention. Considering the physiological effects of music intervention on the mother and fetus, this type of intervention needs more time and further researches.
